# Coumarin Derivatives Act as Novel Inhibitors of Human Dipeptidyl Peptidase III: Combined In Vitro and In Silico Study

**DOI:** 10.3390/ph14060540

**Published:** 2021-06-05

**Authors:** Dejan Agić, Maja Karnaš, Domagoj Šubarić, Melita Lončarić, Sanja Tomić, Zrinka Karačić, Drago Bešlo, Vesna Rastija, Maja Molnar, Boris M. Popović, Miroslav Lisjak

**Affiliations:** 1 Faculty of Agrobiotechnical Sciences Osijek, Josip Juraj Strossmayer University of Osijek, 31000 Osijek, Croatia; maja.karnas@fazos.hr (M.K.); domagoj.subaric@fazos.hr (D.Š.); drago.beslo@fazos.hr (D.B.); vesna.rastija@fazos.hr (V.R.); miroslav.lisjak@fazos.hr (M.L.); 2 Faculty of Food Technology Osijek, Josip Juraj Strossmayer University of Osijek, 31000 Osijek, Croatia; melita.loncaric@ptfos.hr (M.L.); maja.molnar@ptfos.hr (M.M.); 3 Division of Organic Chemistry and Biochemistry, Ruđer Bošković Institute, 10000 Zagreb, Croatia; sanja.tomic@irb.hr (S.T.); zrinka.karacic@irb.hr (Z.K.); 4 Department of Field and Vegetable Crops, Faculty of Agriculture, University of Novi Sad, 21000 Novi Sad, Serbia; boris.popovic@polj.uns.ac.rs

**Keywords:** dipeptidyl peptidase III, coumarin derivatives, inhibitor, molecular modeling, metalloproteinase

## Abstract

Dipeptidyl peptidase III (DPP III), a zinc-dependent exopeptidase, is a member of the metalloproteinase family M49 with distribution detected in almost all forms of life. Although the physiological role of human DPP III (hDPP III) is not yet fully elucidated, its involvement in pathophysiological processes such as mammalian pain modulation, blood pressure regulation, and cancer processes, underscores the need to find new hDPP III inhibitors. In this research, five series of structurally different coumarin derivatives were studied to provide a relationship between their inhibitory profile toward hDPP III combining an in vitro assay with an in silico molecular modeling study. The experimental results showed that 26 of the 40 tested compounds exhibited hDPP III inhibitory activity at a concentration of 10 µM. Compound **12** (3-benzoyl-7-hydroxy-2H-chromen-2-one) proved to be the most potent inhibitor with IC_50_ value of 1.10 μM. QSAR modeling indicates that the presence of larger substituents with double and triple bonds and aromatic hydroxyl groups on coumarin derivatives increases their inhibitory activity. Docking predicts that **12** binds to the region of inter-domain cleft of hDPP III while binding mode analysis obtained by MD simulations revealed the importance of 7-OH group on the coumarin core as well as enzyme residues Ile315, Ser317, Glu329, Phe381, Pro387, and Ile390 for the mechanism of the binding pattern and compound **12** stabilization. The present investigation, for the first time, provides an insight into the inhibitory effect of coumarin derivatives on this human metalloproteinase.

## 1. Introduction

Dipeptidyl peptidase III (DPP III) is a zinc-hydrolase that cleaves dipeptides sequentially from the N-terminal of different bioactive peptides [1]. As a member of the metalloproteinase family M49, DPP III distribution is detected in almost all forms of life [2]. Human DPP III is a very well-characterized member of this family in terms of biochemistry, structural biology and computational chemistry [3,4,5,6,7,8,9,10]. Due to the relative non-specificity of the peptide substrates as well as the lack of selective inhibitors of the metallopeptidases of the M49 family, the physiological substrates of DPP III have not been accurately identified, and its fundamental physiological role has not been precisely determined. However, it is assumed that it is involved in post-proteasomal intracellular protein catabolism [3], defense against oxidative stress [11,12] mammalian pain modulatory system [4,13], malignant processes [14,15,16,17] and blood pressure regulation [18,19,20]. Because of its involvement in biological processes, hDPP III has become interesting to investigate as a potential drug target. To obtain information on the mechanism of action of hDPP III, the influence of selected mutations on the enzyme activity was tested, and research was conducted to find potential inhibitors of this mammalian metalloproteinase [21,22,23]. It has recently been shown that newly synthesized guanidiniocarbonylpyrrole–fluorophore conjugates could be used for enzyme sensing and bio-activity inhibiting (theragnostic) studies of DPP III [24]. In search of new inhibitors from natural sources, we previously reported that luteolin has the best inhibitory effect against hDPP III (IC50 = 22 μM) of the 17 flavonoids tested, and that the number and exact distribution of –OH groups on the flavonoid core is important for their inhibitory properties [25]. The latest study of the biological activity of cornelian cherry fruit extracts showed inhibitory activity against hDPP III with bioactive constituent pelargonidin 3-robinobioside with the best binding energy [26]. Coumarin or 2H-chromen-2-one and its derivatives represent an important group of oxygen-containing heterocycles with benzopyrone skeleton [27]. They can be isolated from plant material [28] or synthesized [29]. Coumarin derivatives possess various beneficial biological activities, for example, anticoagulant, anticancer, analgesic, anti-inflammatory, bactericidal, antifungal, anticonvulsant, anti-hypertensive, muscle relaxant, antioxidant, etc. [28]. It is known that coumarins exhibit an inhibitory effect on the enzymes such as acetylcholinesterase, β-secretase, and monoamine oxidase [30]. Studies have also shown that simple coumarin derivatives influence the activity of some zinc-dependent metalloproteinases [31,32,33].

Because of all mentioned above as well as our efforts to find new hDPP III inhibitors, we report the investigation of 40 structurally different coumarin compounds and provide a relationship between their inhibitory profile toward hDPP III combining in vitro assay with Quantitative Structure–Activity Relationship (QSAR) analysis. Additionally, docking and MD simulations were conducted to explore the mechanism of the most potent inhibitor binding into the active site of hDPP III.

## 2. Results and Discussion

### 2.1. DPP III Inhibitory Activity

In the current study, we evaluated forty various coumarin compounds for their inhibitory potential towards hDPP III. Results in Table 1 showed that substituted 3-acetyl-2H-chromen-2-ones with a bromo group at the C6 position (compound **1**) was the most active with the inhibition rate of 28.5%, while the presence of a hydroxyl group at the same position on compound **2** reduced (12.8%) the inhibitory potential. Shifting a hydroxyl group to C7 in **4** resulted in a slightly increased inhibitory potential (16.2%) as compared to **2** while the presence of diethylamino group at this position in compound **3** was found to be inactive. Additionally, compounds **5** and **6** which possess a hydroxyl and ethoxy group at C8 were completely inactive at the concentration of 10 μM. Compound **7** bearing unsubstituted 3-acetyl-2H-chromen-2-one showed a weak (7.8%) inhibitory activity.

The most potent inhibitory potential of substituted 3-benzoyl-2H-chromen-2-ones was obtained with compound **12** (100.0%) where the hydroxyl group is present at C7. However, the substitution of C7 with the benzoyl (**11**) and methoxy (**13**) group caused a decrease in hDPP III inhibitory (22.8% and 16.5%, respectively) activity. Moderate (67.5%) to weak (4.4%) enzyme inhibition was observed with compounds **10** and **8** which possess a hydroxyl and chloro group at the C6 position, respectively. Compound **9** with a bromo group at the C6 and C8 as well as compound **14** with an ethoxy group on C8 did not exhibit inhibition effects on enzyme activities. Unsubstituted 3-benzoyl-2H-chromen-2-one (**15**) showed only a weak inhibitory activity (9.6%) as compared to **12**.

Of the seven substituted 2-oxo-2H-chromene-3-carbonitriles tested, only compounds **21** and **18** which only differ with hydroxyl group positions (C8 and C6, respectively) moderately inhibited enzyme with an inhibition rate of 62.6% and 44.6%, respectively. In the case where the ethoxy group is at the C8 position (**22**), no inhibitory activity was observed. Furthermore, the methoxy substituent at the C6 position (**17**) gave an inhibition rate of 19.8% while its presence at the C7 position (**20**) was found to be inactive. Substitution of bromo group at C6 in **16** and benzoyl group at C7 in **19** showed almost similar inhibitory potential (7.9% and 7.1%, respectively). Unsubstituted 2-oxo-2H-chromene-3-carbonitrile (**23**) was not effective in inhibiting hDPP III.

Four of seven substituted 3-acetyl-2H-chromen-2-ones: **24**–**27** possess different groups but in the same C6 position. Compound **27** with hydroxyl group and **26** with dihydroxyamino group were found to be more active (66.0% and 59.7% respectively) than the compounds **25** (20.1%) and **24** (not active) with the chloro and bromo group, respectively. Interestingly, dibromo substituents at C6 and C8 (**28**) increased inhibitory potential (29.4%) as compared to mono substituted analog (**24**). Compounds **29** and **30** which possess methoxy group at C7 and ethoxy group at C8, respectively as well as unsubstituted 3-acetyl-2H-chromen-2-one (**31**) did not exhibit inhibitory potential.

Among the substituted methyl 2-oxo-2H-chromene-3-carboxylates, only **36** containing a hydroxyl group at C7 completely inhibited enzymatic activity. Changing the methoxy group at the same position (**37**) completely reduced inhibitory potential. The compound **34** with dihydroxyamino and compound **33** with hydroxyl group at C6 position were found to be more efficient in the inhibitory potential (23.5% and 21.2%, respectively) as compared to the methoxy (9.9%) and bromo (6.5%) substituted analogs **35** and **32**, respectively. A very weak inhibitory potential (2.3%) was found for unsubstituted methyl 2-oxo-2H-chromene-3-carboxylate (**38**). Similarly, 7-hydroxycoumarin (**39**) and coumarin (**40**) exhibited a strong decrease (2.1% and not active, respectively) in the inhibitory potential towards hDPP III.

**Figure 1:**
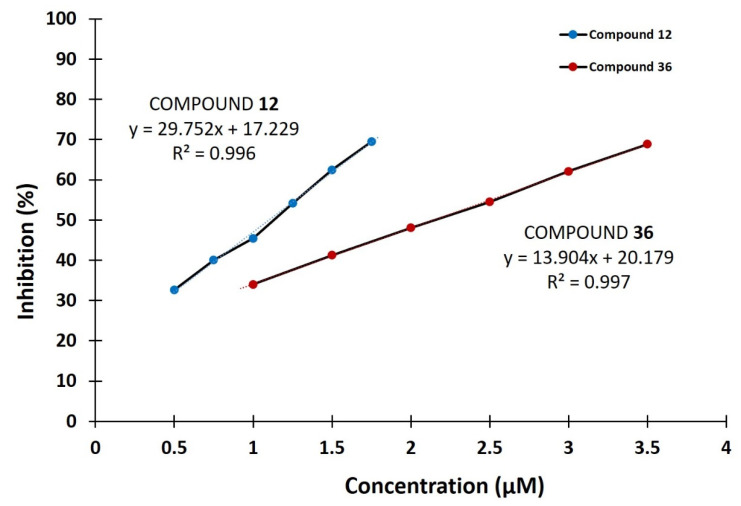
IC_50_ value determination of compounds **12** and **36** against hDPP III. Data points represent the average values of three determinations.

From the above analysis, it can be concluded that the best inhibitory potential had substituted 3-benzoyl-2H-chromen-2-one (**12**) and methyl 7-hydroxy-2-oxo-2*H*-chromene-3-carboxylate (**36**) containing a hydroxyl group at position C7, where they completely inhibited the enzyme at the concentration of 10 μM with IC_50_ values of 1.10 ± 0.05 μM and 2.14 ± 0.06 μM, respectively (Table 1 and Figure 1). Additionally, comparing the structures of derivatives **12** and **36** with compounds **39** and **40** suggests that in addition to the presence of the hydroxyl group at the C7 position, the exact presence of particular substituents at the C3 position is important for increasing the inhibitory activity of tested coumarin derivatives. Furthermore, when the hydroxyl group is at the C6 and C8 positions, the compounds mostly show moderate inhibitory activity. Coumarin derivatives with a substituted bromo, chloro, and benzoyl group showed lower inhibitory potential compared to C6 hydroxy analogs. Finally, compounds with a dihydroxyamino group at the C7 position had moderate enzyme inhibition while most coumarin derivatives with a substituted methoxy and diethylamino group showed no inhibitory activity against hDPP III.

### 2.2. Results of the QSAR Analysis

The best QSAR model obtained for hDPP III inhibition is:

log % hDPP III inh. = −4.03 + 1.82 (0.58) *EEig05x* + 1.46 (0.49) *Mor10u* + 0.49 (0.36) *nArOH* (1)

where *EEig05x* is an edge-adjacency index descriptor weighted by edge degrees, *Mor10u* is a 3D-MoRSE descriptor (unweighted) and *nArOH* is the number of aromatic hydroxyl groups.

**Figure 2:**
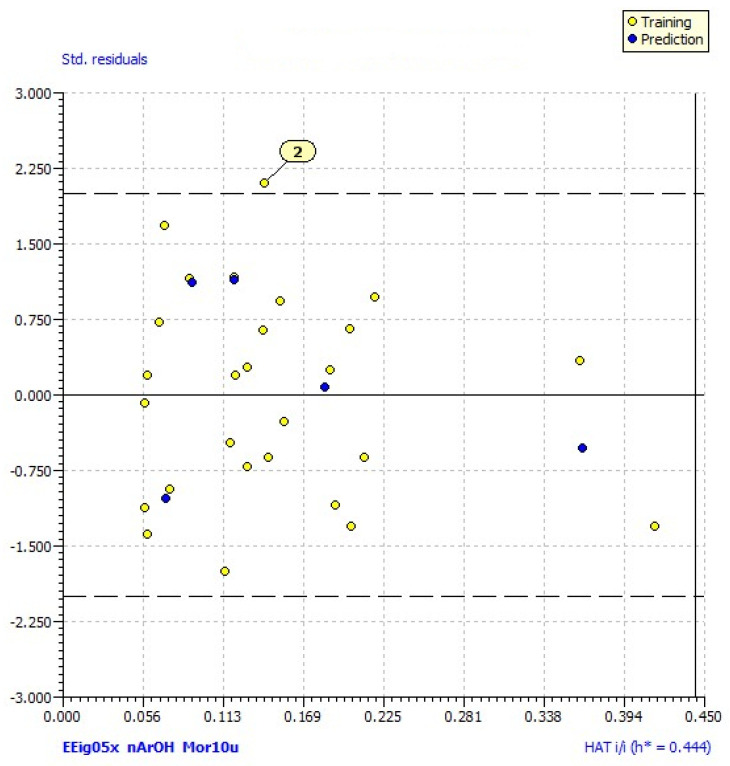
Williams plot (plot of standardized residuals vs. leverages (*h*) for each compound) of applicability domain of the QSAR model for hDPP III inhibition calculated by model 1. The warning leverage (h* = 0.444) is defined as *3p′/n* (*n* is the number of training compounds, and *p′* the number of model adjustable parameters).

The model satisfied the threshold for the fitting and internal validation criteria [34], but Williams plot revealed one outlier, compound **2** (MolID in QSARINS: **24**) as shown in Figure 2. After the exclusion of this compound from the dataset, the subsequent analysis produced the improved QSAR model:

log % hDPP III inh. = −4.07 + 1.85 (0.59) *EEig05x* + 1.60 (0.52) *Mor10u* + 0.56 (0.39) *nArOH* (2)

The variables in Equations (1) and (2) are listed in order of relative importance by their standardized regression coefficient (*β*, written in brackets). The statistical parameters for both models are given in Table 2. The values of the descriptors included in the models are given in the Supplementary Materials (Table S1). The values of log % hDPP III inh.; both experimentally obtained and calculated by Equation (2) are presented in Table 1 and Table S1.

The collinearity of the descriptors in the model was evaluated with a correlation matrix (Table 3) to exclude the possibility that the improved model is overfitted (correlation coefficient *R* ≤ 0.7). Furthermore, low collinearity was verified with the low value of *K_xx_* and Δ*K* being ≥0.05. The model satisfied fitting and internal validation criteria: *R^2^* and *R^2^_adj_* ≥ 0.60; *CCC_tr_* ≥ 0.85; *RMSE* and *MAE* close to zero; *RMSE_tr_* < *RMSE_cv_*; *Q^2^_LOO_* ≥ 0.50 (with *R^2^* − *Q^2^* being low); high value of *F* (Table 2). The value of the cross-validated correlation coefficient (*Q^2^_LOO_* = 0.710) shows that model 2 has a good internal prediction power. The robustness of the improved model was confirmed with both *R^2^_Yscr_* and *Q^2^_Yscr_* values < 0.2, and *R^2^_Yscr_* > *Q^2^_Yscr_* [34]. Model 2 also satisfied the following external validation criteria: *R^2^_ext_* ≥ 0.60; low differences between *RMSE_tr_* and *RMSE_ext_* as well as between *MAE_tr_* and *MAE_ext_* and between *CCC_tr_* and *CCC_ext_*; *Q^2^_F1_*, *Q^2^_F2_*, and *Q^2^_F3_* ≥ 0.60; *r^2^_m_* average ≥ 0.60 and *r^2^_m_* difference ≤ 0.20 indicating that this model could be used for external prediction

The Williams plot for model (2) showed no compounds outside the applicability domain of the model.

The descriptors from the best model were more closely observed to gain insight into the factors that contribute to the inhibitory activity of tested compounds. The first variable in Equation (2) with a high positive contribution is descriptor *EEig05x*, 5th eigenvalue from edge adjacency matrix weighted by edge degrees (the bond order of the various edges). It belongs to the edge-adjacency topological indices derived from the edge adjacency matrix, which encodes the connectivity between graph edges, and is derived from an H-depleted molecular graph of molecules. These descriptors are sensitive to the size, shape, branching, and cyclicity of molecules [35,36]. It is shown that compounds with relatively higher values of this descriptor tend to exhibit higher inhibition of hDPP III. This indicates that compounds with larger, aromatic, and substituents with a higher number of double or triple bonds may exhibit enhanced inhibition. Similar conclusions were also drawn in previous work [37].

The second variable, *Mor10u*, belongs to the 3D-MoRSE (Molecule Representation of Structures based on Electron diffraction) group of descriptors. It has a scattering parameter *s* = 9 Å^−1^ and since it is unweighted, treats all atoms equally [38]. The positive coefficient of *Mor10u* in Equation (2) indicates the importance of the three-dimensional arrangement of all atoms in a molecule and their pairwise distances. Compounds with higher inhibitory activity tend to have more positive values of this descriptor. Since larger molecules, with larger interatomic distances, have higher MoRSE descriptor values, this confirms the above conclusion about the *EEig05x* descriptor that larger molecules are more active.

The third variable in the equation is *nArOH*, a descriptor from the functional group counts that represents the number of aromatic hydroxyls [38]. The positive coefficient in Equation (2) indicates that the presence of aromatic hydroxyl groups contributes to the inhibition of hDPP III. This is in accordance with an earlier study, where the presence of hydrophilic regions (i.e., hydroxyl groups) in flavonoids increased their inhibitory activity against hDPP III [25]. Williams plot revealed compound **2** as an outlier since it had a high predicted residual (predicted value in model (1) was significantly higher than the experimentally obtained). The presence of the hydroxyl group at position 6 might be the reason for the increase in the estimated value according to Equation (1), as well as the high value of *Mor10u* (Table S1). However, this model equation does not consider the presence of substituents at position 3, such as the -COCH_3_ group in this case, that may have a negative effect on inhibition since compounds with this substituent exhibited relatively low inhibition values (Table 1).

**Figure 3:**
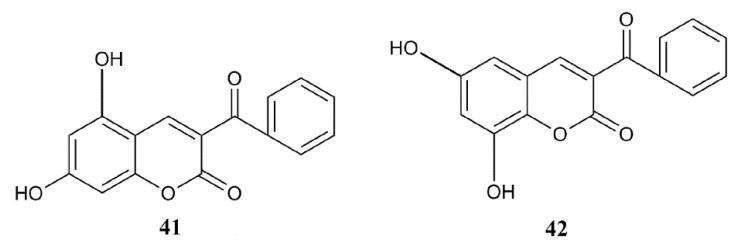
Structures of proposed compounds with possible enhanced inhibition of hDPP III.

Based on the conclusions given in the QSAR analysis, structures of two modified compounds (**41**, **42**) with possible improved activity are proposed, log (% inh. hDPP III) 3.08 and 3.01, respectively (Figure 3). Values of their calculated descriptors, as well as predicted inhibitory activities of the proposed compounds calculated using Equation (2) are given in the Supplementary Materials (Table S1). Since their calculated values exceed 100% of inhibition, these compounds could be potent inhibitors at concentrations lower than 10 µM. Both compounds possess a benzoyl group at the position C-3, and two hydroxyl groups at the position C-5 and C-7 (**41**), and at the position C-6 and C-8 (**42**). Improved calculated inhibition can be attributed to the introduction of an aromatic substituent and additional hydroxyl groups, as indicated by QSAR analysis.

### 2.3. Docking

**Figure 4:**
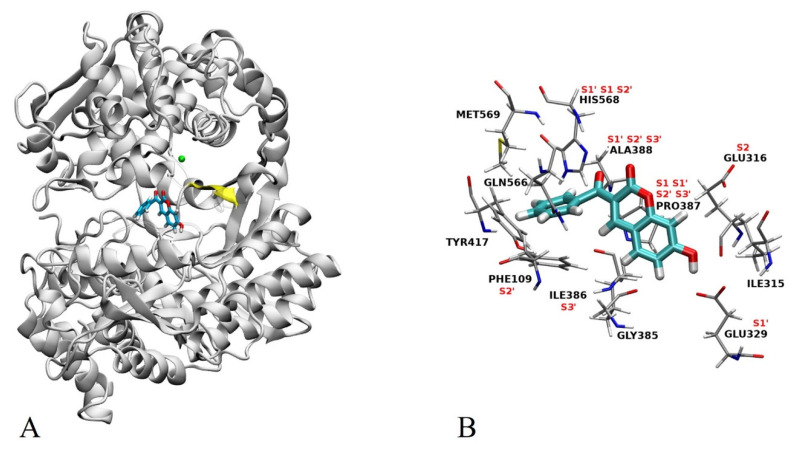
(**A**) Best docking pose for compound **12** in the inter-domain cleft of hDPP III. Compound **12** is shown in stick representation, the lower β sheet is colored yellow, and zinc cation is represented as a green sphere. (**B**) Potential interactions of compound **12** with amino acid residues of hDPP III as presented in the 2D scheme (Figure S1). Substrate binding subsites S1, S1′, S2, S2′ and S3′ are indicated.

In order to obtain further information on the possible interactions of the most active compound (**12**) with a semi-closed form of hDPP III, we combined docking with MD simulations. The best results regarding AutoDock Vina binding energy (−8.6 kcal mol^−1^) of enzyme–ligand complex predict that compound **12** binds to the inter-domain cleft, near the lower β sheet (residues 389–393) (Figure 4A) and enzyme active site, with the minimum distance between the catalytic Zn cation and **12** (oxygen atom at C2 of coumarin core) being ~7 Å. In this complex, the position of compound **12** closely resembles the substrate position in the hDPP III active site [9] which is accommodated similarly to the opioid peptides in the enzyme binding pocket [5]. Namely, binding of **12** into the inter-domain cleft is accompanied by its interactions mostly with amino acid residues of the hDPP III S1, S1′, S2, S2′ and S3′ substrate binding subsite (Figure S1 and Figure 4B).

### 2.4. MD Simulations

To prove the reliability of the best docking result, the binding mode of compound **12** in complex with hDPP III was investigated by productive MD simulations using the AMBER16 software package. Simulations of complex were performed in three replicates, each 300 ns long, and used for comparison. Dynamic behavior, protein, and ligand stability during simulations were analyzed by root mean square deviation (RMSD), while the analysis of the intermolecular interactions during MD simulations included hydrogen bonding (H-bond), native contacts, and Gibbs free energy. Representative structures of the complex were used to describe intermolecular interactions in more detail.

#### 2.4.1. RMSD Profile

**Figure 5:**
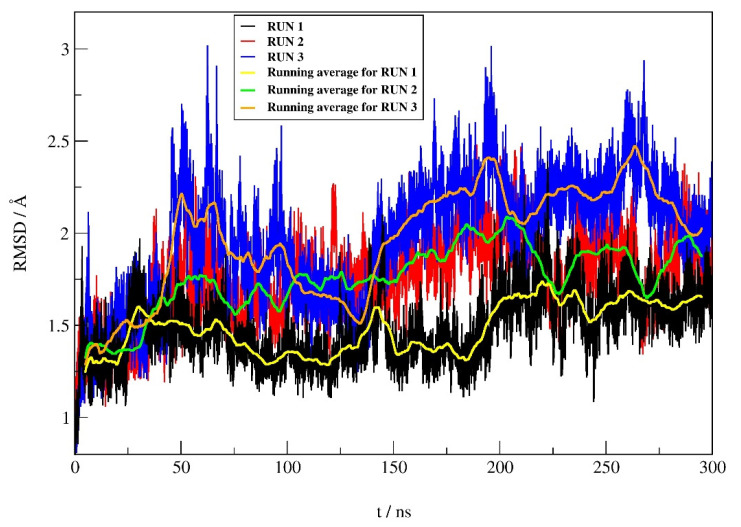
RMSD profile of the protein backbone atoms obtained during 300 ns of MD simulations.

**Figure 6:**
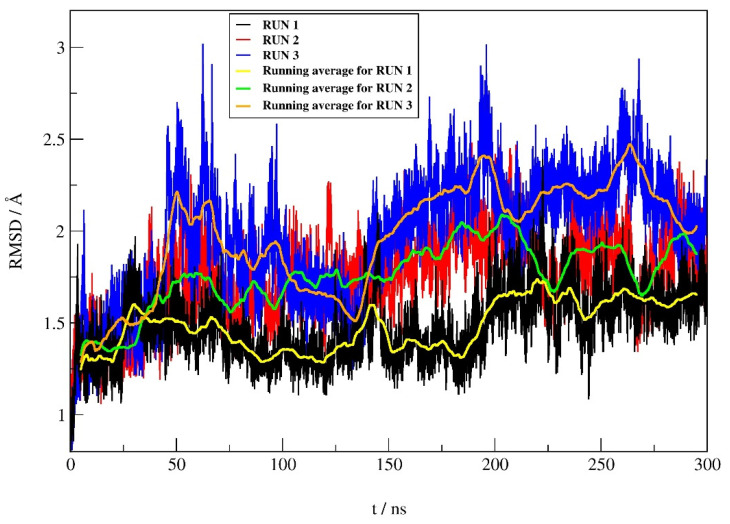
RMSD profile for the heavy atoms (hydrogen atoms were not considered) of compound **12** obtained from MD simulations of complex.

The RMSD profiles (Figure 5) calculated during the simulations for the protein backbone atoms show similar protein stability in all three runs, with only slightly higher protein stability in run 1 (average RMSD ± SD of 1.48 ± 0.18 Å, 1.76 ± 0.23 Å, and 1.96 ± 0.34 Å for run 1, 2, and 3, respectively). According to the RMSD values for the heavy atoms of compound **12** between replicates, it can be seen (Figure 6) that the stability of **12** is better in run 1 compared to the other two replicates. The average RMSD ± SD were 0.37 ± 0.15 Å, 0.46 ± 0.12 Å and 0.54 ± 0.15 Å, for run 1, run 2 and run 3, respectively.

#### 2.4.2. Hydrogen Bond Analysis

Hydrogen bond analysis was undertaken to investigate the stability and occupancy of hydrogen bonds between compound **12** and the key residues of the binding site of hDPP III. The results of trajectory H-bonds analysis for all three replicates are listed in Table S2.

**Figure 7:**
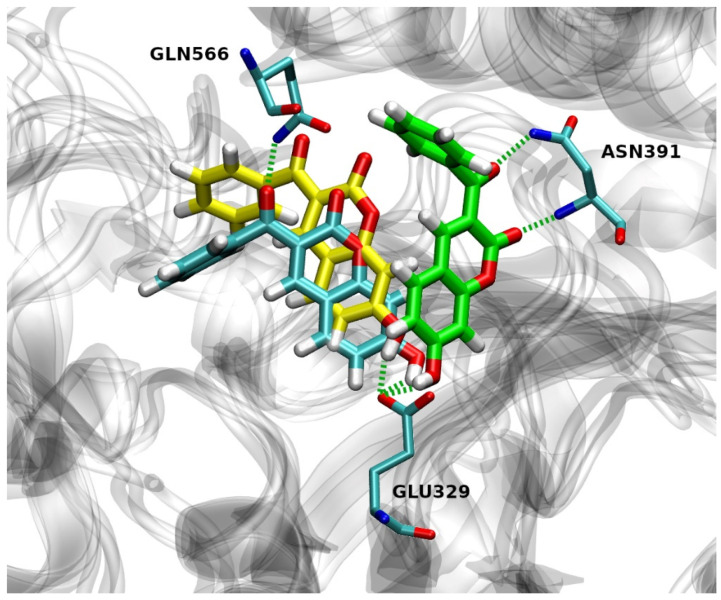
Overlay of the hDPP III with compound **12** in its preferable binding mode after 300 ns of MD simulation for run 1 (cyan), run 2 (green) and run 3 (yellow). Hydrogen bonds are depicted as green dashed lines.

In run 1, there were two H-bonds formed during the MD process with the occupation time >10% (Figure 7). The first H-bond is formed by the OE2 atom of Glu329 and the H-O4 of **12** with an occupation time of 99%, and the second one is formed by the H-NE2 of Gln566 and the O3 of **12** with an occupation time of 23%. Additionally, atom O2 of compound **12** forms H-bond with H-NE2 of Gln566 and H-OH of Tyr318 with the occupation time of 5% and 4%, respectively.

Three hydrogen bonds were formed during the simulations in run 2 with the occupation time >10% (Figure 7). The first one is formed by the OE1 atom of Glu329 and the H-O4 of **12** with an occupation time of 85%. The second and third H-bond was formed between the H-N and H-NH2 of Asn391 and the O2 and O3 of **12** with the occupation time of 17% and 13%, respectively. One H-bond with an occupation time of 6% is formed by the H-ND2 of Asn391 and the O2 of compound **12.**

Only one hydrogen bond (with the occupation time > 10%) was formed during the simulations in run 3 by the OE2 atom of Glu329 and the H–O4 of **12** with an occupation time of 99% (Figure 7). Moreover, atom O3 of **12** forms H-bond with H–OH of Tyr417 and H-NE2 of His568, both with the occupation time of 4%, while atom O2 of **12** forms H-bond with H-NE2 of His568 with the occupation time of 3%. It is worthwhile to note that Glu329 and Gln568 are found to be constituents of hDPP III S1, S1′and S2′ substrate-binding subsites [5]. Besides this, His568 and Asn491 are highly conserved residues among known DPP IIIs [39].

#### 2.4.3. Native Contacts

To further investigate the interactions of hDPP III and compound **12**, we calculated the relative occupancy of native contacts during the MD simulations. Relative occupancy of native contacts is defined as the sum of fractions of native contacts during the trajectory for each residue pair relative to the total number of native contacts involved with that pair. Native contacts were defined as a distance between the atoms of enzyme residues and atoms of compound **12** within a distance cutoff of 5 Å. The fractions of native contacts were calculated using the *nativecontacts* command in the CPPTRAJ module.

**Figure 8:**
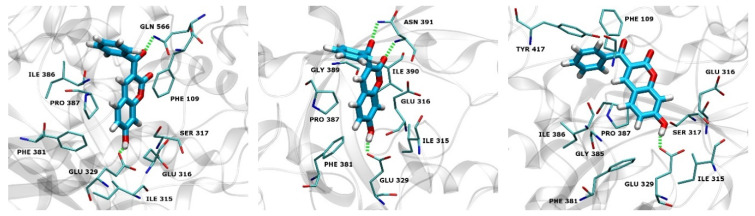
hDPP III residues involved in native contacts and H-bond formation with compound **12** for run 1 (**left**), run 2 (**middle**) and run 3 (**right**). H-bonds are depicted as green dashed lines, and compound **12** as light blue sticks.

**Figure 9:**
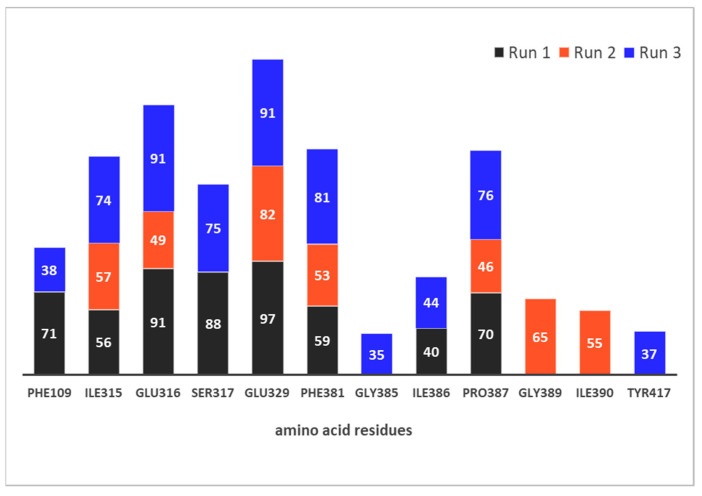
Native contacts between hDPP III residues and compound **12** with relative occupancy of more than 30% during 300 ns MD simulations.

Figure 8 and Figure 9 depict the protein residues that were involved in forming native contacts with a relative occupancy of more than 30% in the run 1, 2, and 3 of the complex throughout the simulation time. In all three replicates, the protein forms native contacts with **12** through residues Ile315, Glu316, Glu329, Phe381, and Pro387, which indicates that these residues could be quite important for the stabilization of the complex. In runs 1 and 3, protein additionally forms native contacts with compound **12** through Phe 109, Ser317, and Ile386. The remaining contacts are formed in runs 2 and 3 through residues Gly389 and Ile390, and Gly385 and Tyr417, respectively. Some of the listed amino acid residues such as Glu316, Ser317, Glu329, Ile386, Pro387 and Tyr417 were found to contribute to the binding of synthetic inhibitors into the active site of hDPP III [22,24,39].

#### 2.4.4. Types of Intermolecular Interactions

A detailed analysis of different types of intermolecular interactions between replicates was performed using the Discovery Studio Visualizer. For this purpose, the extracted complex structures obtained from trajectory after 300 ns of MD simulations were optimized and used as representative. According to Figures S2–S4 representing 2D schemes of the intermolecular interaction types, compound **12** forms almost the same number of interactions with amino acid residues in all three replicates. However, in run 1, the largest number of interactions is formed between the coumarin core of **12** and the hDPP III, which is not the case in the other two replicates, where the benzoyl group of compound **12** also interacts with the amino acid residues. The above differences in the distributions of intermolecular interactions of **12** and amino acid residues relate mainly to the van der Waals interactions as shown in Figures S2–S4.

Comparing the other types of intermolecular interactions between replicates, the 2D schemes show that Glu329 and Pro387 form H-bonds and π-alkyl interactions with coumarin core in all three replicates, while additional H-bond and π-alkyl are formed with Asn391 and Ile390 in run 2, respectively. Additionally, in run 1, coumarin core forms π–π stacked interaction with Phe109 and amide π stacked interaction with Ile386. The benzoyl group of compound **12** forms one π-donor H-bond with Ile386, one π-alkyl with Ala567, and one π–π shaped interaction in run 1, run 2 and run 3, respectively.

#### 2.4.5. MM-GBSA Free Energy Calculations

MM-GBSA calculations were used to obtain quantitative estimates of the free binding energies of compound **12** in the complex with the hDPP III for all three replicates. According to the results given in Table 4, the electrostatic contribution (ΔE_ele_) is the most important to the ΔG_bind_ for complex in run 1 and run 2. This is in accordance with the results of MD simulations because in run 1 and run 2, compound **12** formed two and three H-bonds (with the occupation time > 10%), respectively, relative to run 3 where only one H-bond is formed. Another important contribution to the ΔG_bind_ of the complexes is the van der Waals interactions (E_vdw_) with values similar in all three replicates. These results are in line with the observed similarities of the native contacts formed during the simulations, especially with residues Ile315, Phe381, and Pro387 in all three replicates. The unfavorable polar solvation contribution (E_GB_) was slightly higher for complex in run 2 compared to runs 1 and 3, while the favorable nonpolar contribution (E_SA_) had similar values for all three replicates.

From the estimated values of ΔG_bind_ between replicates, it can be concluded that the reason for the highest free binding energy in run 1 (−26.36 kcal mol^−1^) is in the more favored E_GB_ and E_ele_ contribution compared to those in run 2 and run 3, respectively.

## 3. Material and Methods

### 3.1. Synthesis of Coumarin Derivatives

Synthesis and characterization of the coumarin derivatives were performed as described previously [40]. Briefly, series of coumarin derivatives were synthesized via Knoevenagel condensation starting from various substituted salicylaldehydes and ethyl acetoacetate (series 1; compounds **1**–**7**), ethyl benzoylacetate (series 2; compounds **8**–**15**), ethyl cyanoacetate (series 3; compounds **16**–**23**), diethyl malonate (series 4; compounds **24**–**31**), and dimethyl malonate (series 5, compounds **32**–**38**). 7-hydroxycoumarin and coumarin (compounds **39** and **40** respectively) were purchased from Sigma Chemical Co. (St. Louis, MO, USA). The structures of the tested compounds are presented in Table 1.

### 3.2. Heterologous Expression and Purification of Human DPP III

C-terminally truncated human DPP III was expressed and purified as described by Kumar et al. [7]. Briefly, C-terminally truncated hDPP III gene on a pET28-MHL plasmid, with an N-terminal His-tag and a TEV protease cleavage site fusion, was expressed in BL21-CodonPlus (DE3) RIL E. coli strain using 0.25 mM IPTG for induction of expression at 18 °C and 130 rpm shaking. After 20 h, cells were centrifuged and frozen at −20 °C until purification. Bacterial cells were lysed by a combination of lysozyme lysis and sonication, and the lysate was centrifuged to precipitate the cell debris. A brief DNase I treatment was performed before centrifugation to reduce the viscosity caused by DNA released by lysis. The lysate was purified on a Ni-NTA column (5 mL prepacked His-trap FF, GE Healthcare) using a buffer system with 50 mM Tris HCl, pH = 8.0, 300 mM NaCl, and increasing imidazole concentrations: 10 mM for lysis, 20 mM for wash and 300 mM for elution buffer. Fractions with hDPP III were pooled and incubated with TEV protease to remove the His-tag. hDPP III was recovered using flow-through affinity chromatography (TEV protease is His-tagged), and additionally purified on a 16/60 Superdex-200 gel-filtration column (GE Healthcare). Main fractions were pooled and desalted. Aliquots of protein in 20 mM Tris HCl buffer pH = 7.4 were stored at −80 °C until use. SDS PAGE of the purified enzyme was presented in Figure S5.

### 3.3. Assay of Human DPP III Activity

Purified hDPP III (1.5 nM) was preincubated with coumarin derivatives (10 µM) first for 5 min at 25 °C and then for 10 min at 37 °C in 50 mM Tris-HCl buffer, pH 7.4. The enzymatic reaction was started with Arg_2_-2NA (40 μM) as a substrate, and after the 15 min incubation at 37 °C the reaction was stopped and the absorbance was measured using the spectrophotometric method described before [41]. Percentage enzyme inhibition (% inh.) was calculated by comparing the enzymatic activity without (control activity), and with inhibitor (inhibited activity) using the following formula:

% inh. = [(control activity−inhibited activity)/(control activity)] × 100%

The IC_50_ values of selected compounds (**12** and **36**) were determined by the linear regression of the percentage of enzyme inhibition against the increasing concentrations (0.5–3.5 μM) of coumarin derivatives. The IC_50_ value is defined as the concentration of an inhibitor that caused a 50% reduction in the enzyme activity under assay conditions. The stock solutions (8 mM) of coumarin derivatives were freshly prepared in dimethyl sulfoxide and diluted with 50 mM Tris-HCl buffer, pH 7.4 buffer before assay of enzymatic activity.

### 3.4. Molecular Modeling

#### 3.4.1. QSAR Analysis

Randomly ordered structures of 38 coumarin derivatives, coumarin, and 7-hydroxycoumarin (40 compounds in total) were drawn and optimized using the MM+ molecular mechanics force field [42]. Afterward, the structures were also subjected to geometry optimization using the PM3 semi-empirical method [43], using the Polak–Ribiere algorithm, until the root-mean-square gradient (RMS) was 0.1 kcal/(Åmol). Drawing and optimization of structures were performed in Avogadro 1.2.0. (University of Pittsburgh, Pittsburgh, PA, USA) [44].

Descriptor calculation for the resulted minimum energy conformations of compounds was performed with Parameter Client (Virtual Computational Chemistry Laboratory, an electronic remote version of the Dragon program) [45]. Logarithmic values of experimentally obtained hDPP III inhibition percentages were taken as response values. The generation and validation of QSAR models were performed using QSARINS 2.2.4 (University of Insubria, Varese, Italy) [46].

In order to reduce a large number of calculated descriptors, constant and semi-constant descriptors, i.e., those with a constant value for more than 85% of compounds, and descriptors that were too intercorrelated (>95%) were rejected by QSARINS. The final number of remaining descriptors was 514. Due to the high number of inactive compounds (14), 8 of them were randomly chosen and excluded from the dataset. A genetic algorithm (GA) was used to generate the best model. The number of descriptors in the multiple linear regression equation was limited to three. The splitting of compounds into the training set (n = 27 molecules) and test set (n = 5 molecules) was performed by activity sampling [47]. Compounds were ranked by their activities (from the most active to the least active compound) and then divided into five groups of the approximately same size. One compound was selected randomly from each group and assigned to the test set. The models were validated by the internal cross-validation performed using the “leave-one-out” (LOO) and Yscrambling method [46]. The following evaluation criteria were included: coefficient of determination (*R*^2^), adjusted coefficient of determination (*R^2^_adj_*), cross-validated correlation coefficient (*Q^2^*_LOO_), inter-correlation among descriptors (*K*_xx_), the difference of the correlation among the descriptors and the descriptors plus the responses (Δ*K*), the standard deviation of regression (*s*), Fisher ratio (*F*), root-mean-square error (*RMSE*); LOO cross-validated root-mean-square error (*RMSE_cv_*), concordance correlation coefficient (*CCC*), LOO cross-validation concordance correlation coefficient (*CCC_cv_*), mean absolute error of the training set (*MAE*), mean absolute error of the internal validation set (*MAE_cv_*), and LOO cross-validated predictive residual sum of squares (*PRESS_cv_*). QSAR model robustness was tested using the Y-randomization test, giving *R^2^_Yscr_* and *Q^2^_Yscr_* values [34]. External validation parameters included the coefficient of determination of the test set (*R*^2^*_ext_*), external validation set root-mean-square error (*RMSE_ext_*), external validation set concordance correlation coefficient (*CCC_ext_*), external validation set mean absolute error (*MAE_ext_*), predictive squared correlation coefficients (*Q^2^_F1_*, *Q^2^_F2_*, *Q^2^_F3_*) and the average value of squared correlation coefficients between the observed and LOO predicted values of the compounds with and without intercept (*r^2^_m_*) [48].

To identify the possible outliers and compounds out of the warning leverage (h*) in a model, a leverage plot (plot of standardized residuals vs. leverages (*h*); the Williams plot) was used. The warning leverage is generally defined as *3p′/n* (*n* being the number of training compounds, and *p′* the number of model adjustable parameters [49]. Outliers in the Williams plot are compounds that have values of standardized residuals higher than two standard deviation units.

#### 3.4.2. Preparation of the Complex Structure

The complex between the enzyme and compound **12** was built using the semi-closed conformation of hDPP III obtained earlier [8] by MD simulations of the structure available in the Protein Data Bank (PDB code: 3FVY), since it has been proved that this is the most preferable enzyme form in water solution [50]. Before the docking procedure, the protonation of histidines was checked according to their ability to form hydrogen bonds with neighboring amino acid residues. All Glu and Asp residues are negatively charged (−1) and all Arg and Lys residues are positively charged (+1), as expected at physiological conditions. AutoDock Vina 1.1.2 [51] was used to search for the best pose of the ligand to the enzyme active site. The docking site was defined as a cubical grid box with dimensions 75 × 75 × 75 Å^3^ and the center placed on the Zn^2+^. Docking simulation was done with the standard 0.375 Å resolution and 20 conformations were generated. The complex with the best AutoDock Vina docking score was chosen for the productive MD simulations. Parameterization of the complex structure was performed by the AMBERTools16 modules *antechamber* and *tleap* using General Amber Force Field (GAFF) [52] and ff14SB [53] force fields to parameterize the ligand and the protein, respectively. For the zinc cation, Zn^2+^, new hybrid bonded-nonbonded parameters were used from our previous work [54]. The complex was dipped into the truncated octahedral box filled with TIP3P water molecules with a margin distance of 11 Å. Besides water molecules, 24 sodium ions were added to neutralize the system and placed in the vicinity of charged amino acids at the protein surface.

#### 3.4.3. Molecular Dynamics Simulations

Before the productive MD simulations, the complex was energy-minimized in three cycles to eliminate or reduce the energy constraints. Firstly, 1500 steps of minimization were performed, where the first 450 steps were of the steepest descent method, and the rest was the conjugate gradient. Both the protein atoms and the metal were constrained using a harmonic potential of force constant 32 kcal/(mol Å^2^), to equilibrate water molecules. Secondly, 2500 steps were performed and only the first 470 steps of steepest descent were used. The metal and protein backbone were constrained with 32 kcal/(mol Å^2^). Finally, in the third cycle, the same number of minimization steps was as in the first cycle, and both protein backbone and metal were constrained with 10 and 32 kcal/(mol Å^2^), respectively. Next, the minimized system was heated from 0 to 300 K during 30 ps using a canonical ensemble (NVT), and then equilibrated 80 ps during which the initial constraints on the protein and the metal ion were used. This was followed by another equilibration stage of 100 ps, during which the initial constraints on the protein and the metal ion were removed and the water density was adjusted. The time step during the periods of heating and the water density adjustment was 1 fs. The equilibrated system was then subjected to 300 ns of the productive MD simulations (in three replicates) at constant temperature and pressure (300 K and 1 atm) using the NPT ensemble, without any constraints. The temperature was held constant using Langevin dynamics with a collision frequency of 1 ps^−1^. Bonds involving hydrogen atoms were constrained using the SHAKE algorithm [55]. Simulations of the complex were performed within the AMBER16 software package [56]. The time step used for the productive MD simulations was set to 2 fs and the trajectory files were collected every 10 ps for the subsequent analysis. Trajectory analysis and the binding free energies (ΔG_bind_) evaluation was performed by the CPPTRAJ module and MMPBSA.py script, respectively, from the AmberTools16 program package and examined visually using VMD 1.9.3 [57] and Discovery Studio Visualizer, version 20.1.0.19295 (BIOVIA, San Diego, CA, USA) software [58].

## 4. Conclusions

In summary, the potential hDPP III inhibitory activity of a series of coumarin derivatives was investigated for the first time by combining in vitro and in silico approaches. Compound **12** (3-benzoyl-7-hydroxy-2H-chromen-2-one) was found to be the most potent inhibitory molecule with IC_50_ value of 1.10 μM. The productive MD simulations indicate that H-bonds between the 7-OH group of compound **12** and the carboxyl group of Glu329 as well as van der Waals interactions with Ile315, Ser317, Phe381, Pro387, and Ile390 are important for the mechanism of binding. According to the results of QSAR and binding mode analyses, two new compounds with possible improved activity were proposed. The discovery of coumarin derivatives as hDPP III inhibitors may provide new clues to the relationship between the chemical structure and biological activity of these naturally occurring compounds and their derivatives, and provide guidelines for the development of novel coumarin scaffolds as potent inhibitors of this mammalian metalloproteinase.

## Figures and Tables

**Table 1:** Structures of analysed compounds, values of experimentally determined inhibition of hDPP III (at 10 µM concentration of compounds) and calculated logarithmic values of the % inhibition of hDPP III.

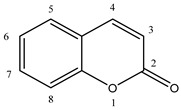				
Compound No.	Substituents	DPP III Inh. (%)	Log (% hDPP III Inh.) exp.	Log (% DPP III Inh.) Calc. *
**1**	3-acetyl; 6-bromo	28.5	1.45	1.29
**2**	3-acetyl; 6-hydroxy	12.8	1.11	Excl.
**3**	3-acetyl; 7-diethylamino	NA	0.00	-
**4**	3-acetyl; 7-hydroxy	16.2	1.21	1.70
**5**	3-acetyl; 8-ethoxy	NA	0.00	0.35
**6**	3-acetyl; 8-hydroxy	NA	0.00	-
**7**	3-acetyl	7.8	0.89	1.01
**8**	3-benzoyl; 6-chloro	4.4	0.64	1.01
**9**	3-benzoyl; 6,8-dibromo	NA	0.00	-
**10**	3-benzoyl; 6-hydroxy	67.5	1.83	1.95
**11**	3-benzoyl; 7-benzoyl	22.8	1.36	1.18
**12**	3-benzoyl; 7-hydroxy	100.0 (1.10 ± 0.05 µM)	2.00	1.89
**13**	3-benzoyl; 7-methoxy	16.5	1.22	0.78
**14**	3-benzoyl; 8-ethoxy	NA	0.00	-
**15**	3-benzoyl	9.6	0.98	0.65
**16**	3-cyano; 6-bromo	7.9	0.90	0.98
**17**	3-cyano; 6-methoxy	19.8	1.30	0.83
**18**	3-cyano; 6-hydroxy	44.6	1.65	1.81
**19**	3- cyano; 7-benzoyl	7.1	0.85	1.04
**20**	3-cyano; 7-methoxy	NA	0.00	-
**21**	3- cyano; 8-hydroxy	62.6	1.80	1.67
**22**	3-cyano; 8-ethoxy	NA	0.00	0.17
**23**	3-cyano	NA	0.00	-
**24**	3-ethoxycarbonyl; 6-bromo	NA	0.00	-
**25**	3-ethoxycarbonyl; 6-chloro	20.1	1.30	0.93
**26**	3-ethoxycarbonyl; 6-dihydroxyamino	59.7	1.78	1.49
**27**	3-ethoxycarbonyl; 6-hydroxy	66.0	1.82	1.76
**28**	3- ethoxycarbonyl; 6,8-dibromo	29.4	1.47	1.82
**29**	3-ethoxycarbonyl; 7-methoxy	NA	0.00	0.26
**30**	3-ethoxycarbonyl; 8-ethoxy	NA	0.00	0.37
**31**	3- ethoxycarbonyl	NA	0.00	-
**32**	3- methoxycarbonyl; 6-bromo	6.5	0.81	0.78
**33**	3-methoxycarbonyl; 6-dihydroxyamino	21.2	1.33	1.46
**34**	3-methoxycarbonyl; 6-hydroxy	23.5	1.37	1.35
**35**	3-methoxycarbonyl; 6-methoxy	9.9	1.00	0.64
**36**	3-methoxycarbonyl; 7-hydroxy	100.0 (2.14 ± 0.06 µM)	2.00	1.50
**37**	3-methoxycarbonyl; 7-methoxy	NA	0.00	0.56
**38**	3-methoxycarbonyl	2.3	0.35	0.59
**39**	coumarin	NA	0.00	−0.37
**40**	7-hydroxycoumarin	2.1	0.33	0.49

NA, no activity; Excl.; excluded as outlier; -, excluded from initial dataset; * Calculated by quantitative structure-activity relationship (QSAR) equation: log % hDPP III inh. = −4.07 + 1.85 (0.59) *EEig05x* + 1.60 (0.52) *Mor10u* + 0.56 (0.39) *nArOH*; numbers in brackets represent IC_50_ values.

**Table 2:** The statistical parameters for QSAR models.

Statistical Parameters	Model 1	Model 2
*N_tr_*	27	26
*N_ex_*	5	5
*R^2^*	0.746	0.796
*R^2^_adj_*	0.713	0.768
*s*	0.352	0.323
*F*	22.565	28.572
*K_xx_*	0.210	0.190
Δ*K*	0.201	0.215
*RMSE_tr_*	0.325	0.297
*MAE_tr_*	0.276	0.253
*CCC_tr_*	0.855	0.886
*Q^2^_LOO_*	0.650	0.710
*RMSE_cv_*	0.381	0.354
*MAE_cv_*	0.326	0.302
*PRESS_cv_*	3.923	3.258
*CCC_cv_*	0.807	0.844
*R^2^_Yscr_*	0.115	0.121
*Q^2^_Yscr_*	−0.236	−0.244
*RMSE_ext_*	0.292	0.295
*MAE_ext_*	0.255	0.275
*R^2^_ext_*	0.795	0.785
*CCC_ext_*	0.868	0.873
*Q^2^_F1_*	0.783	0.778
*Q^2^_F2_*	0.780	0.776
*Q^2^_F3_*	0.794	0.798
*r^2^_m_* average	0.614	0.653
*r^2^_m_* difference	0.186	0.174
Applicability domain		
*N* outliers	1 (2)	-
*N* out of app. domain	-	-

*N_tr_* (number of compounds in training set); *N_ex_* (number of compounds in test set); LOO (leave-one-out); *R*^2^ (coefficient of determination); *R*^2^_adj_ (adjusted coefficient of determination); *s* (standard deviation of regression); *F* (Fisher ratio); *K_xx_* (global correlation among descriptors); Δ*K* (global correlation among descriptors); *RMSE_tr_* (root-mean-square error of the training set); *MAE_tr_* (mean absolute error of the training set); *CCC_tr_* (concordance correlation coefficient of the training set); *Q^2^_LOO_* (cross-validated explained variance); *RMSE_cv_* (root-mean-square error of the training set determined through the cross validated method); *MAE_cv_* (mean absolute error of the internal validation set); *PRESS_cv_* (cross-validated predictive residual sum of squares); *CCC_cv_* (concordance correlation coefficient test set using cross validation); *R*^2^*_Yscr_* (Y-scramble correlation coefficients); *Q*^2^*_Yscr_* (Y-scramble cross-validation coefficients); *RMSE_ext_* (root-mean-square error of the external validation set); *MAE_ext_* (mean absolute error of the external validation set); *R*^2^*_ext_* (coefficient of determination of external validation set); *CCC_ext_* (concordance correlation coefficient of the test set); *Q*^2^*_F_*_1_, *Q*^2^*_F_*_2_, *Q*^2^*_F_*_3_ (predictive squared correlation coefficients); *r^2^_m_* average (average value of squared correlation coefficients between the observed and leave-one-out predicted values of the compounds with and without intercept); *r^2^_m_* difference (absolute difference between the observed and leave-one-out predicted values of the compounds with and without intercept).

**Table 3:** Correlation matrix (with correlation coefficient values *R*) for descriptors used in Equation (2).

Descriptor	*EEig05x*	*Mor10u*	*nArOH*
*EEig05x*	1.000		
*Mor10u*	−0.264	1.000	
*nArOH*	−0.129	0.489	1.000

**Table 4:** Binding free energy (kcal mol^−1^) of the complexes obtained during the last 5 ns of MD simulations for all three replicates.

Energy Component	Run 1	Run 2	Run 3
E_vdw_	−28.33	−27.44	−26.16
E_ele_	−30.55	−30.76	−23.34
E_GB_	35.94	41.26	34.69
E_SA_	−3.44	−4.06	−3.41
ΔG_gas_	−58.88	−58.20	−49.50
ΔG_solv_	32.50	37.20	31.28
ΔG_bind_	−26.36	−20.98	−18.22

E_vdw_—van der Waals potential energy; E_ele_—electrostatic energy; E_GB_—polar solvation energy; E_SA_—nonpolar solvation energy; ΔG_gas_—gas phase free energy; ΔG_solv_—solvation free energy; ΔG_bind_—binding free energy.

## Data Availability

The data presented in this study are available on request from the corresponding author.

## Supplementary Materials

The following are available online at https://www.mdpi.com/article/10.3390/ph14060540/s1. Table S1: The values of the descriptors included in QSAR model equation (2): log % hDPP III inh. = −4.07 + 1.85 (0.59) *EEig05x* + 1.60 (0.52) *Mor10u* + 0.56 (0.39) *nArOH*; Table S2: Detailed analysis of hydrogen bonds between compound **12** (LIG) and hDPP III residues during MD simulations for run 1, 2 and 3 obtained by *hbond* command in CPPTRAJ module; Figure S1: 2D diagram of compound **12** interactions with the hDPP III residues for the best docking pose; Figure S2: 2D diagram of compound **12** interactions with the hDPP III residues for run 1; Figure S3: 2D diagram of compound **12** interactions with the hDPP III residues for run 2; Figure S4: 2D diagram of compound **12** interactions with the hDPP III residues for run 3; Figure S5: SDS-PAGE demostrating purity of hDPP III sample on a 10% gel: M. PageRuler Prestained protein marker. with 72 kDa band in red; lane 1. hDPP III sample after affinity chromatography; lanes 2–5. fractions of the main hDPP III peak after gel-filtration.
