# Supplementary material for: Coumarin Derivatives Act as Novel Inhibitors of Human Dipeptidyl Peptidase III: Combined In Vitro and In Silico Study
Source: Pharmaceuticals (Basel). 2021 Jun 5;14(6):540. doi: 10.3390/ph14060540 (PMC8229952; doi:10.3390/ph14060540)
Supplement: Supplementary file 1 [file pharmaceuticals-14-00540-s001.zip › pharmaceuticals-1224476-supplementary.pdf]

Supplementary material

Coumarin derivatives act as novel inhibitors of human dipeptidyl peptidase III: combined in vitro and in silico study.

Dejan Agić[1*], Maja Karnaš[1], Domagoj Šubarić[1], Melita Lončarić[2], Sanja Tomić[3], Zrinka Karačić[3], Drago Bešlo[1], Vesna Rastija[1], Maja Molnar[2], Boris M. Popović[4] and Miroslav Lisjak[1].

1. Faculty of Agrobiotechnical Sciences Osijek. Josip Juraj Strossmayer University of Osijek. 31000 Osijek. Croatia; dejan.agic@fazos.hr (D.A.); maja.karnas@fazos.hr (M.K.); domagoj.subaric@fazos.hr (D.Š.); vesna.rastija@fazos.hr (V.R.); drago.beslo@fazos.hr (D.B.); miroslav.lisjak@fazos.hr (M.L.)
2. Faculty of Food Technology Osijek. Josip Juraj Strossmayer University of Osijek. 31000 Osijek. Croatia; melita.loncaric@ptfos.hr (M.L.); maja.molnar@ptfos.hr (M.M.)
3. Divison of Organic Chemistry and Biochemistry. Ruđer Bošković Institute. 10000 Zagreb. Croatia; sanja.tomic@irb.hr (S.T.); zrinka.karacic@irb.hr (Z.K.)
4. Department of Field and Vegetable Crops. Faculty of Agriculture. University of Novi Sad. 21000 Novi Sad. Serbia; boris.popovic@polj.uns.ac.rs (B.P.)

*Correspondence: dejan.agic@fazos.hr (D.A.)

Table S1 The values of the descriptors included in QSAR model equation (2): log % hDPP III inh. = - 4.07 + 1.85 (0.59) *EEig05x* + 1.60 (0.52) *Mor10u* + 0.56 (0.39) *nArOH*

MolID in QSARINS	Compound	*EEig05x*	*Mor10u*	*nArOH*	log(% inh DPP III) exp.	log(% inh DPP III) calc. by Eq 2
**1**	31	2.864	-0.442	0	0.00	-
**2**	15	3.015	-0.538	0	0.98	0.65
**3**	11	3.474	-0.735	0	1.36	1.18
**4**	24	3.010	-0.387	0	0.00	-
**5**	34	3.000	-0.434	1	1.37	1.35
**6**	27	3.010	-0.188	1	1.82	1.76
**7**	25	3.010	-0.359	0	1.30	0.93
**8**	9	3.295	-0.450	0	0.00	-
**9**	37	2.951	-0.522	0	0.00	0.56
**10**	29	2.962	-0.719	0	0.00	0.26
**11**	35	3.000	-0.529	0	1.00	0.64
**12**	30	2.867	-0.543	0	0.00	0.37
**13**	14	3.211	-0.852	0	0.00	-
**14**	33	3.001	-0.013	0	1.33	1.46
**15**	26	3.011	-0.009	0	1.78	1.49
**16**	7	2.857	-0.127	0	0.89	1.01
**17**	23	2.868	-0.355	0	0.00	-
**18**	6	2.857	0.116	1	0.00	-

**19**	21	2.874	-0.086	1	1.80	1.67
**20**	4	2.916	-0.117	1	1.21	1.70
**21**	3	2.929	-0.525	0	0.00	-
**22**	1	2.966	-0.080	0	1.45	1.29
**23**	16	3.027	-0.348	0	0.90	0.98
**24**	2	2.966	-0.041	1	1.11	excluded as outlier
**25**	18	3.027	-0.174	1	1.65	1.81
**26**	20	2.984	-0.492	0	0.00	-
**27**	17	3.027	-0.442	0	1.30	0.83
**28**	5	2.857	-0.541	0	0.00	0.35
**29**	22	2.875	-0.676	0	0.00	0.17
**30**	38	2.863	-0.397	0	0.35	0.59
**31**	19	3.312	-0.639	0	0.85	1.04
**32**	36	2.948	-0.277	1	2.00	1.50
**33**	12	3.239	-0.374	1	2.00	1.89
**34**	32	3.000	-0.439	0	0.81	0.78
**35**	8	3.210	-0.540	0	0.64	1.01
**36**	13	3.239	-0.716	0	1.22	0.78
**37**	10	3.210	-0.304	1	1.83	1.95
**38**	28	3.273	-0.102	0	1.47	1.82
**39**	39	2.344	-0.399	0	0.00	-0.37
**40**	40	2.410	-0.289	1	0.33	0.49
**41**		3.340	-0.091	2		3.08
**42**		3.295	-0.088	2		3.01

- Inactive compounds excluded from QSAR

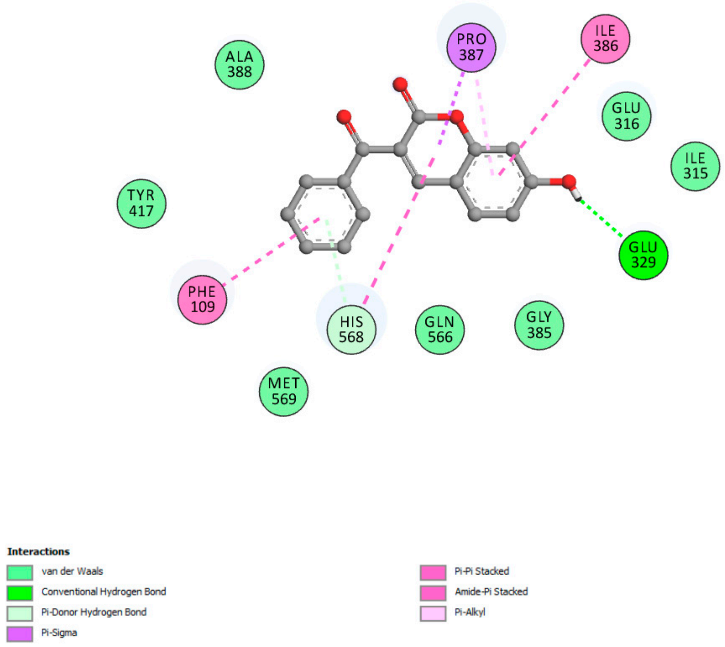

Figure S1 2D diagram of compound **12** interactions with the hDPP III residues for the best docking pose.

Table S2 Detailed analysis of hydrogen bonds between compound **12** (LIG) and hDPP III residues during MD simulations for run 1. 2 and 3 obtained by *hbond* command in CPPTRAJ module.

RUN	Acceptor	Donor H	Donor	Frames	Fraction	AvgDist	AvgAngle
1	GLU_329@OE2	LIG@H1	LIG@O4	29794	0.9931	2.6228	165.3042
	LIG@O3	GLN_566@HE22	GLN_566@NE2	6794	0.2265	2.8563	155.2041
	LIG@O2	GLN_566@HE22	GLN_566@NE2	1476	0.0492	2.8484	147.1674
	LIG@O2	TYR_318@HH	TYR_318@OH	1241	0.0414	2.7842	160.8479
2	GLU_329@OE1	LIG@H1	LIG@O4	25524	0.8508	2.6332	164.773
	LIG@O2	ASN_391@H	ASN_391@N	5150	0.1717	2.8898	156.9507
	LIG@O3	ASN_391@HD22	ASN_391@ND2	4028	0.1343	2.8492	157.513
	LIG@O2	ASN_391@HD22	ASN_391@ND2	1788	0.0596	2.8756	155.3083
3	GLU_329@OE2	LIG@H1	LIG@O4	29832	0.9944	2.6313	164.7486
	LIG@O3	TYR_417@HH	TYR_417@OH	1180	0.0393	2.7920	157.2633
	LIG@O3	HIE_568@HE2	HIE_568@NE2	1078	0.0359	2.8720	151.3918
	LIG@O2	HIE_568@HE2	HIE_568@NE2	911	0.0304	2.8777	153.9514

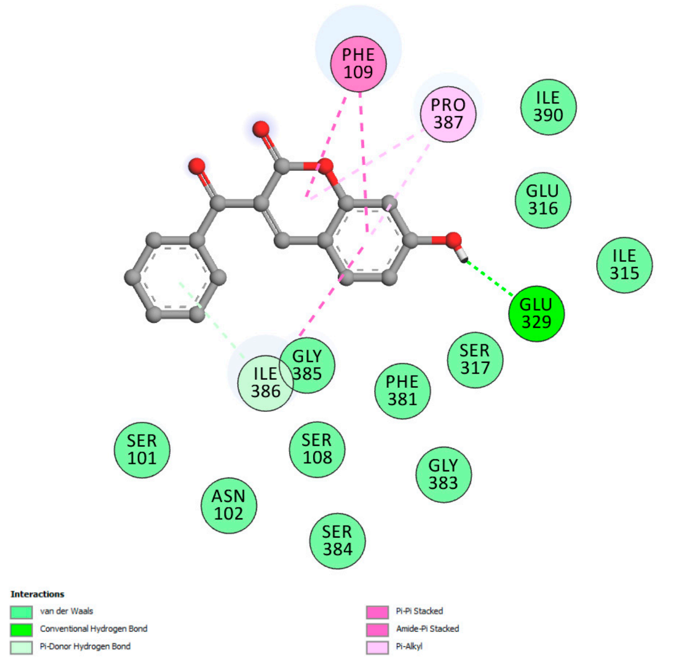

Figure S2 2D diagram of compound **12** interactions with the hDPP III residues for run 1

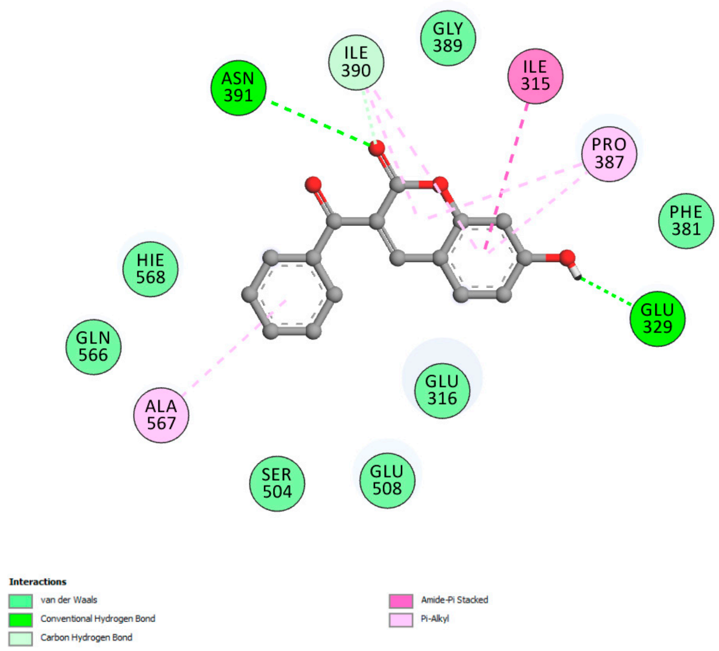

Figure S3 2D diagram of compound **12** interactions with the hDPP III residues for run 2

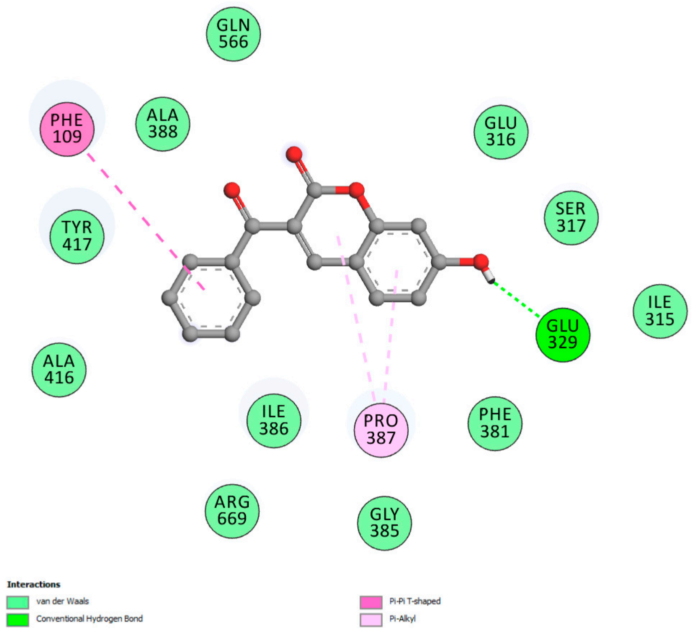

Figure S4 2D diagram of compound **12** interactions with the hDPP III residues for run 3

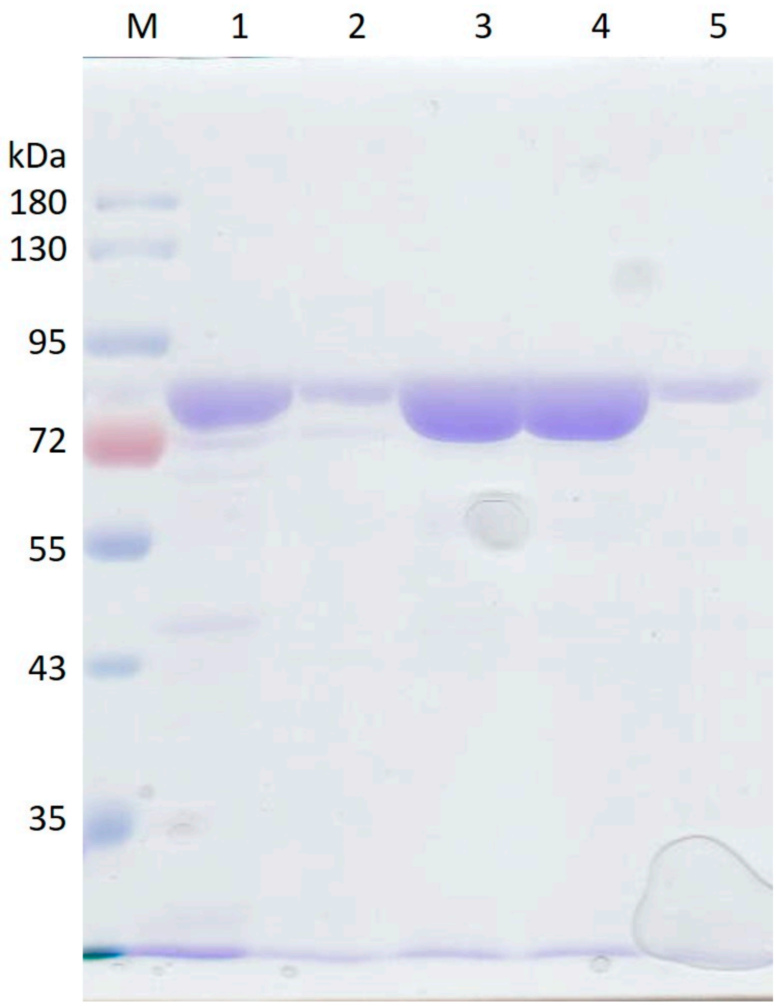

Figure S5 SDS-PAGE demostrating purity of hDPP III sample on a 10% gel: M. PageRuler Prestained protein marker. with 72 kDa band in red; lane 1. hDPP III sample after affinity chromatography; lanes 2-5. fractions of the main hDPP III peak after gel-filtration.
